# Multi-omics approach reveals the contribution of *OsSEH1* to rice cold tolerance

**DOI:** 10.3389/fpls.2022.1110724

**Published:** 2023-01-13

**Authors:** Shuang Gu, Jia Zhuang, Zhe Zhang, Wanchun Chen, Hai Xu, Minghui Zhao, Dianrong Ma

**Affiliations:** Rice Research Institute/Collaborative Innovation Center for Genetic Improvement and High Quality and Efficiency Production of Northeast Japonica Rice in China, Shenyang Agricultural University, Shenyang, China

**Keywords:** *OsSEH1*, cold tolerance, metabolome, transcriptome, ROS, phenylpropanoid

## Abstract

As low environmental temperature adversely affects the growth, development and geographical distribution, plants have evolved multiple mechanisms involving changing physiological and metabolic processes to adapt to cold stress. In this study, we revealed that nucleoporin-coding gene *OsSEH1* was a positive regulator of cold stress in rice. Physiological assays showed that the activity of antioxidant enzymes showed a significant difference between *osseh1* knock-out lines and wild type under cold stress. Metabolome analysis revealed that the contents of large-scale flavonoids serving as ROS scavengers were lower in *osseh1* mutants compared with wild type under cold stress. Transcriptome analysis indicated that the DEGs between *osseh1* knock-out lines and wild type plants were enriched in defense response, regulation of hormone levels and oxidation-reduction process. Integration of transcriptomic and metabolic profiling revealed that *OsSEH1* plays a role in the oxidation-reduction process by coordinately regulating genes expression and metabolite accumulation involved in phenylpropanoid and flavonoid biosynthetic pathway. In addition, Exogenous ABA application assays indicated that *osseh1* lines had hypersensitive phenotypes compared with wild type plants, suggesting that *OsSEH1* may mediate cold tolerance by regulating ABA levels.

## Introduction

Rice (*Oryza sativa* L.), a staple food crop that feeds over half of the world’s population, originates from tropical and subtropical regions and is sensitive to cold stress (Sasaki and Burr, 2000). Cold stress has been identified as one of the main factors restricting the growth, development, production, and geographical distribution of rice (Sperotto et al., 2018). In Northeast China, the growth and development of rice are seriously affected by cold stress at the seedling and booting stages (Sun et al., 2022). As a result of severe cold disasters, rice production in Northeast China was drastically reduced by 42% in 1972 and 37% in 1976 (Yang et al., 2017a). Therefore, it is important to mine cold-tolerant genes and elucidate their regulatory mechanisms for national food security and sustainable agricultural development.

Plants exposed to cold stress suffer from wilting, discoloration, leaf margin drying, accelerated aging, incomplete ripening, and even death (Zhang et al., 2016). Various evaluation indices were used to reflect cold tolerance at the seedling stage, including survival rate, leaf withering degree, proline content, soluble sugar content, and the activity of antioxidant enzymes (Han et al., 2020). The survival rate and degree of leaf withering reflect the external phenotype of seedlings under cold stress. As cytoplasmic osmotic pressure regulators, proline and soluble sugars can enhance cold tolerance (Ma et al., 2009; Gaveliene et al., 2014). Under normal conditions, reactive oxygen species (ROS) are well known to act as molecular signals or secondary messengers that regulate plant growth at lower concentrations (Mittler, 2017). However, under cold conditions, overaccumulation of ROS degrades polyunsaturated lipids, oxidizes proteins, and damages cells (Han et al., 2017). Antioxidant enzymes play an important role in maintaining cellular redox homeostasis. The activity of antioxidant enzymes, such as superoxide dismutase (SOD), peroxidase (POD), ascorbate peroxidase (APX), and catalase (CAT), reflects the ability of plants to mitigate ROS under cold stress (Noctor and Foyer, 1998).

Plants synthesize a variety of secondary metabolites from the amino acid phenylalanine, including benzenoids, coumarins, flavonoids, hydroxycinnamates, and lignin (Vogt, 2010). These compounds are collectively referred to as phenylpropanoids and play an essential role in plant development and plant–environment interactions (Dong and Lin, 2021). For example, lignins are a large group of aromatic polymers that are deposited in the plant cell wall, serving as both structural support and a plant defense mechanism (Boerjan et al., 2003; Vanholme et al., 2019). The synthesis of lignin can be induced by many types of abiotic stressors, such as drought, cold stress, and mineral deficiency, as well as biotic stresses, including infection by fungi, bacteria, or viruses (Moura et al., 2010). Flavonoids, another important class of soluble phenylpropanoids, have long been suggested to have multiple functions in plant development and adaptation to environmental stress (Agati et al., 2012; Agati et al., 2013; Nakabayashi et al., 2014). Under unfavorable conditions, such as UV light, drought, and biotic stress, flavonoids accumulate in plants to protect cells from oxidative damage (Treutter, 2005; Hassan and Mathesius, 2012; Agati et al., 2013; Nakabayashi et al., 2014). Recently, phenylpropanoid responses to environmental temperature in plants, which are considered to play a vital role in cold stress, have gathered more attention (Sudheeran et al., 2018; Zhang et al., 2022).

Cold tolerance is a complex agronomic trait controlled by multiple genes (Guo et al., 2018; Shi et al., 2018). Traditional genetic and molecular analysis has been used to identify major QTLs/genes controlling cold tolerance in rice, including *low-temperature germinability on chromasome 3* (*qLTG3*), *low temperature growth 1* (*LTG1*), *chilling tolerance divergence 1* (*COLD1*), *cold tolerance at booting stage 4a* (*CTB4a*), *basic leucine zipper 73* (*bZIP73*), and *HAN1* (“han” is termed “chilling” in Chinese) (Fujino et al., 2008; Lu et al., 2014; Ma et al., 2015; Zhang et al., 2017; Liu et al., 2018; Liu et al., 2019; Mao et al., 2019). Nuclear pore complexes (NPCs), consisting of multiple nucleoporins (Nups), play vital roles in the exchange of macromolecules, such as RNAs and proteins (Parry, 2015; Yang et al., 2017b). Some studies have suggested that Nups also play an important role in regulating cold tolerance. For instance, *NUP160* was shown to be involved in cold stress responses, since the *nup160* lines impaired the expression of the *CBF3*-*LUC* reporter gene and cold response (*COR*) genes, resulting in hypersensitivity to cold stress (Dong et al., 2006b). In addition, *high expression of osmotically responsive genes 1* (*HOS1*) is considered a negative regulator of cold signaling (Ishitani et al., 1998). The expression of *COR* genes in *hos1* mutants was higher than that in wild-type (WT) plants (Lee et al., 2001). HOS1 was further shown to modulate the protein levels of ICE1 (inducer of CBF expression 1) by ubiquitination to attenuate cold signaling (Lee et al., 2001; Dong et al., 2006a). However, the functions of other nucleoporins in cold signaling remain poorly understood, especially in rice.

Our previous study detected QTLs for cold tolerance at the seedling stage through genome-wide association studies using Ting’s rice core collection (Song et al., 2018). At all these QTLs, a major locus on chromosome 1 explained 27% of phenotypic variance. We subsequently analyzed candidate genes within this locus and noticed that the expression of the nucleoporin-coding gene *OsSEH1* was dramatically induced by cold stress. Hence, our previous results indicate that *OsSEH1* is a potential candidate gene for cold tolerance in rice. However, further characterization of gene function and its regulatory mechanism in response to cold stress in rice requires further investigation. In the current study, we revealed by combining transcriptomic and metabolomic methods that *OsSEH1* regulates many genes and metabolites involved in the phenylpropanoid pathway in response to cold stress. Moreover, we showed that exogenous abscisic acid (ABA) increased the cold tolerance of *osseh1* knockout lines, but had little effect on WT plants. This study advances our understanding of the function of plant nucleoporins in cold stress and provides a potential genetic resource for generating cold-tolerant rice varieties.

## Materials and methods

### Plant materials and growth conditions

Two rice genotypes, wild type MangShuiDao (MSD) and its mutant *osseh1*, were selected for use in this study. MSD is a cold tolerant temperate *Japonica* landrace from Yangtze River region, China. The mutant *osseh1* lines were generated by CRISPR/Cas9 previously. The mutation sites in the *osseh1* knock-out lines were showed in **Supplementary Figure 1**.

Seeds were surface sterilized with 5% (w/v) sodium hypochlorite for 3 min and then soaked in the water at 28°C for 5 days in the dark. The germinated seeds were transferred to 96-well plates and then grown hydroponically in the solution of International Rice Research Institute (IRRI). The 96-well plates were placed in a plant growth chamber (14h-light/10h-dark conditions) with temperatures of 28°C and 25°C for the light and dark conditions, respectively.

For cold stress at the seedling stage, the seedlings of wild type and *osseh1* mutants were used to test the cold tolerance. The seedlings were transferred to a growth chamber at 4°C for 7 days after knowing which plants were able to recover at 28°C for 7 days, and the survival rates were calculated. Cold treatment was treated at the 16^th^ days of rice seedling growth. The sampling time of physiological indicators was 0 h and 48 h after cold stress.

For the germination assay, sterilized seeds were put in the 0 µM, 1 µM, 10 µM, 100 µM or 150 µM ABA. The germination rates were assessed at 0, 36, 48, 60, 72, 84, 96 h. Three replicate assays were conducted with at least 200 seeds each time.

### Measurement of soluble sugar content

The soluble sugar content was measurement was performed according to the previous study (Yoshida et al., 1971) with some modification. Briefly, leaf sample (0.2 g fresh weight) was fixed in 4 ml 80% ethanol. After centrifugation at 5000 × g for 10 min, added 2.5 ml of anthrone to the supernatant (0.5 ml) and kept in a water bath at 40°C for 30 min. After cooling, measure the optical density of the mixture at 625 nm.

### Measurement of proline content

The proline content was determined according to the previous study (Bates et al., 1973) with some modification. Leaf samples (0.5 g) were boiled in 10ml 3% sulfosalicylic acid and then the cooling homogenate was centrifuged at 3000×g for 10 min. The supernatant (1 ml) was treated with 1 ml acetic acid and 2 ml 2.5% ninhydrin, boiled for 1 h, and absorbance was determined at 520 nm.

### Measurement of ROS content

The ROS content was determined by plant ROS enzyme-linked immunity kit (Jiangsu Meimian Industrial Co., Ltd., Yancheng, China) according to the manufacturer’s protocol. Double antibody sandwich method was used in the kit to determine the content of plant ROS in the leaves sample. Purified plant ROS antibodies were placed in the microporous plate to form solid-phase antibodies. Use purified ROS antibody to coat the microplate to prepare solid phase antibody. Add ROS to the microplate coated with monoclonal antibody in turn, and then combine with HRP (horse radish peroxidase) labeled ROS antibody to form antibody antigen enzyme labeled antibody complex. After thorough washing, add substrate TMB (3, 3′,5,5′-Tetramethylbenzidine) for color development. TMB is catalyzed by HRP enzymes to turn blue and converted to the final yellow color by acid. The shade of color was positively correlated with ROS in the sample. The absorbance (OD) was measured at 450nm and the concentration of ROS was calculated by standard curve.

### Measurements for antioxidative enzyme activity

Fresh leaves (about 0.2 g) were ground in cold 2 mL 50 mM PBS solution. Centrifuged homogenate at 8000 r/min for 20 min at 4°C. The supernatant was kept measure the antioxidant enzyme activity. For SOD activity measurement, 50 μL supernatant was added to 5 mL nitroblue tetrazolium (NBT) reaction buffer and then the reaction mixture was kept under 4000 lux lights for 20 min and analyzed at 560 nm using a spectrophotometer (Polle et al., 1989). For POD activity measurement, 50 μL supernatant was added to 5 mL guaiacol reaction buffer and analyzed at 470 nm using a spectrophotometer (Fecht-Christoffers et al., 2006). For CAT activity measurement, 50 μL supernatant was added to 5 mL reaction buffer in the presence of H_2_O_2_ and analyzed at 240 nm using a spectrophotometer (Verma and Dubey, 2003). For APX activity measurement, 50 μL supernatant was added to 5 mL ascorbate reaction buffer and analyzed at 290 nm using a spectrophotometer (Vanacker et al., 1998). All treatments had three biological and three technical replicates.

### Metabolite profiling analysis

Metabolomic profiling was performed using a widely targeted metabolome technology with three independent biological replicates at MetWare Biotechnology Co., Ltd. (Wuhan, China) (Li et al., 2022a). Briefly, the leaves samples were ground using the MM 400 Mixer Mill (Retsch Technology, Haan, Germany) with a zirconia bead for 1.5 min. Then, 100 mg freeze-dried powder was weighted for metabolites extraction with 500 μL of 80% aqueous methanol containing 0.1 mg/L lidocaine at 4°C for 8h. Following centrifugation at 10000g for 15 min, the supernatant was filtered *via* a syringe filter (SCAA-104, 0.22-μm pore size; ANPEL, Shanghai, China) before LC-MS/MS analysis. Quality Control (QC) samples were mixed with all samples to test the reproducibility of the entire experiment. Differentially accumulated metabolites (DAMs) were identified using the t-test < 0.05 and variable importance in projection (VIP) ≥ 1.

### Transcriptome and bioinformatics analysis

RNA-Seq sequencing and analyses were performed by Gene Denovo Biotechnology Co., (Guangzhou, China) as described previously (Li et al., 2022b; Yan et al., 2022). Briefly, total RNA was extracted from the four-leaf stage seedlings using the Trizol Reagent Kit (Invitrogen, Carlsbad, CA, USA) according to the manufacturer’s protocol, with three biological replicates each containing 50 plants. RNA quality and integrity were assessed on the Agilent 2100 Bioanalyzer (Agilent Technologies, Palo Alto, CA, USA). Gene Denovo Biotechnology Co. (Guangzhou, China) performed RNA-Seq sequencing and analyses using the Illumina HiSeq2500 platform. Differentially expressed genes (DEGs) between the *osseh1* mutants and the wild type were identified with false discovery rate (FDR) < 0.05 and absolute fold change ≥ 2. DEGs were then analyzed by Gene Ontology (GO) functions and Kyoto Encyclopedia of Genes and Genomes (KEGG) pathway.

### RNA extraction and RT-qPCR

Total RNA was extracted from the rice seedling leaves using the TransZol Up Plus RNA kit (TransGen Biotech, China) according to the manufacturer’s protocol. RNA quality and concentration were quantified using a NanoDrop 8000 spectrophotometer (Thermo Fisher Scientific). Total RNA was reverse-transcribed to cDNA using One-Step gDNA Removal and cDNA Synthesis SuperMix (TransGen Biotech, China). RT-qPCR was carried out using the PerfectStart Green qPCR SuperMix (TransGen Biotech, China) protocol and the QuantStudio 3 System (Applied Biosystems, USA). Rice *ACTIN1* gene was used as the internal control. Data were analyzed following the relative quantification method (Livak and Schmittgen, 2001). Primer used for RT-qPCR are listed in **Supplementary Table 9**. The experiments were repeated at least three times.

### Exogenous ABA treatment

Hormone treatments were conducted by spraying the leaves of 12-day old seedlings with 1 μM, 10 μM, 100 μM and 150 μM ABA containing 0.1% (v/v) Tween 20 as the surfactant. The seedlings were sprayed with the mixture of different concentration of ABA at 9 a.m. for 3 days and then transferred to a growth chamber at 4°C for 2 days. ABA (Sigma) was dissolved in methanol. The identical volume of the blank methanol containing 0.1% (v/v) Tween20 was used as a mock treatment.

### Endogenous ABA measurement

The ABA content was determined by plant ABA enzyme-linked immunity kit (Jiangsu Meimian Industrial Co., Ltd., Yancheng, China) according to the manufacturer’s protocol. Double antibody sandwich method was used in the kit to determine the content of plant ABA in the leaves sample.

### Statistical analysis

A two-tailed Student’s *t*-test was used to compare the difference of data from two groups, and analysis of variance (ANOVA) one-way comparison followed by Duncan’s tests (p<0.05) was used to compare the difference of data from multiple groups, using SPSS version 26 ((IBM Corp., Armonk, NY, USA).

## Results

### Morphological and physiological characteristics are altered in *osseh1* knock-out lines under cold stress

**Figure 1:**
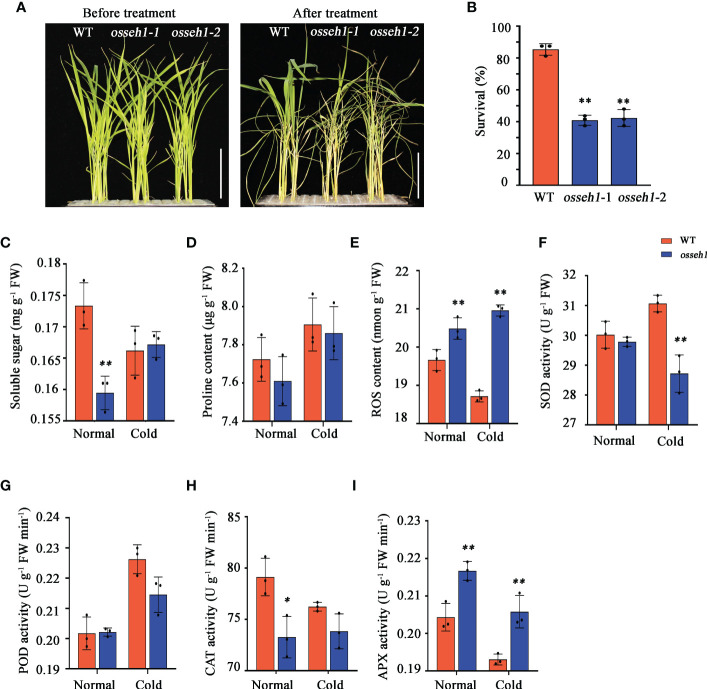
Comparison of morphological and physiological indexes between *osseh1* knock-out lines and WT plants under cold stress. **(A)** Phenotypes of *osseh1* knock-out lines and WT plants under cold tolerance. **(B)** Survival rates of *osseh1* knock-out lines and WT plants recovered for 7 days after cold treatment. Statistical analysis of **(C)** soluble sugar, **(D)** proline content, **(E)** ROS content, **(F)** SOD activity, **(G)** POD activity, **(H)** CAT activity and **(I)** APX activity. Data represents means ± SEM (n = 3). *P < 0.05, **P < 0.01. Scale bars, 5cm. WT, wild type; *osseh1*, knockout lines; ROS, reactive oxygen species. SOD, superoxide dismutase; POD, peroxidase; CAT, catalase; APX, ascorbate peroxidase.

In order to dissect the function of *OsSEH1* in rice, we examined the cold tolerance of wild type (WT) and *osseh1*lines. After 7-day cold treatment and a 7-day recovery, only 41.67% of the *osseh1* seedlings survived, in contrast to 87.5% of the WT plants (**Figures 1A, B**). Further, we measured the physiological parameters of WT plants and *osseh1* knock-out lines before and after cold treatment. The growth performance was assessed by evaluating plant height, root length, shoot fresh and dry weight, root fresh and dry weight. We sampled and measured the parameters at three time points and the first sampling point is the 14^th^ day of the rice seedling (**Supplementary Figure 2A**). For both WT and *osseh1* lines, shoot fresh weight, root fresh weight, shoot dry weight and root dry weight showed similar rising tendency during cold treatment compared with that during normal condition (**Supplementary Figures 2B–E**). However, the plant height and root length of *osseh1* lines was significantly inhibited under cold stress compared with that of WT (**Supplementary Figure 2F, G**).

In addition, we measured soluble sugar content, proline content, ROS content, superoxide dismutase (SOD) activity, peroxidase (POD) activity, ascorbate peroxidase (APX) activity and catalase (CAT) activity in the leaves of WT and *osseh1* lines under normal and cold condition. Compared with the WT lines, the soluble sugar content of *osseh1* lines decreased significantly under normal condition, while there was no significant difference between them under cold stress (**Figure 1C**). The proline content of both WT and *osseh1* lines were increased after cold stress, while there was no significant difference between them under normal and cold condition (**Figure 1D**). Our results also revealed that the ROS content in *osseh1* knock-out lines was significantly higher than that in WT plants, suggesting that *osseh1* lines were subjected to more severe oxidative stress under cold stress (**Figure 1E**). After 2-day 4°C cold stress, the SOD activity in the leaves of *osseh1* is significantly lower than that of WT (**Figure 1F**). The POD activity was no difference between WT and *osseh1* lines under normal and cold condition (**Figure 1G**). The CAT activity of *osseh1* was lower compared to that of WT under normal condition, while there was no significant difference between them under cold condition (**Figure 1H**). The APX activity of *osseh1* lines was significantly higher than that of WT under both normal and cold condition (**Figure 1I**). These results indicated that *OsSEH1* play a role in regulating rice development and physiological characteristics.

### *OsSEH1* regulates a broad range of the metabolite accumulation

**Figure 2:**
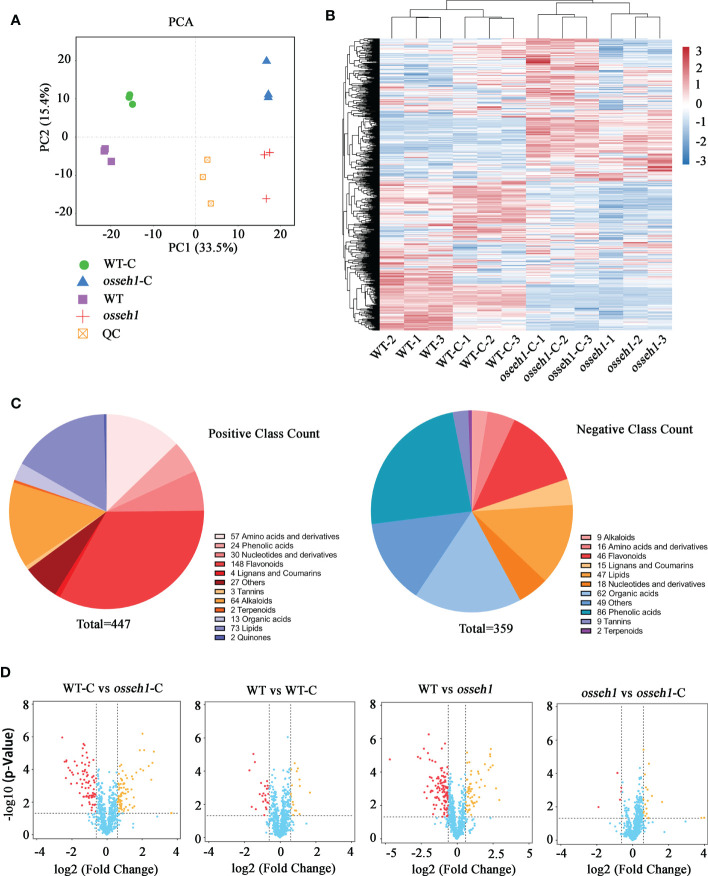
Overview of the metabolite accumulation under normal and cold condition. **(A)** Principal component analysis (PCA) of the metabolic profiles. PC1 and PC2 indicate principal component 1 and principal component 2, respectively. The quality control sample (QC) was prepared by mixing aliquots of all of the samples. **(B)** Heat map visualization of metabolites. The content of each metabolite was normalized to complete linkage hierarchical clustering. **(C)** Classification map of metabolites under positive and negative iron mode. **(D)** Volcano plots of the metabolites from the comparison of WT-C vs. *osseh1*-C, WT vs. WT-C, WT vs. *osseh1* and *osseh1* vs. *osseh1*-C, respectively. WT, WT under normal condition. WT-C, WT under cold treatment. *osseh1*, *osseh1* lines under normal condition. *osseh1*-C, *osseh1* lines under cold treatment.

To reveal the role of *OsSEH1* underlying cold treatment at the metabolic profile, we performed widely targeted metabolomics assay for wild type plants and *osseh1* knock-out mutants. We used an ultra-performance liquid chromatography-tandem mass spectrometry (UPLC-MS) method to identify changes in metabolite levels. Principal component analysis (PCA) indicated that the metabolites of different genotypes and treatments were significantly different (**Figure 2A**). Cluster analysis was also performed, and 12 samples were clearly divided into four groups, indicating significant differences in metabolites among four experiment groups (**Figure 2B**). A total of 806 metabolites were detected with this approach, including 31 different types of substances, among these metabolites, 110 were phenolic acids, 108 were flavonoid metabolites, 75 were organic acids, 73 were amino acids and derivatives, and 54 were free fatty acids (**Supplementary Table 1**; **Supplementary Figure 3**). In positive ion mode, the metabolites were categorized into 12 classes, while in negative ion mode, the metabolites were categorized into 11 classes (**Figure 2C**).

A total of 102 differently accumulated metabolites (DAMs) (32 upregulated and 70 downregulated metabolites) were identified between WT under normal condition and *osseh1* under normal condition (WT vs *osseh1*; variable importance (VIP) scores ≥1 and T-test P<0.05; **Figure 2D**; **Supplementary Table 2**). 81 DAMs (53 upregulated and 28 downregulated metabolites) were identified between the WT under normal condition and WT under cold stress (WT vs WT-C; variable importance (VIP) scores ≥1 and T-test P<0.05; **Figure 2D**; **Supplementary Table 3**). 59 DAMs (54 upregulated and 5 downregulated metabolites) were identified between the *osseh1* under normal condition and *osseh1* under cold stress (*osseh1* vs *osseh1*-C; variable importance (VIP) scores ≥1 and T-test P<0.05; **Figure 2D**; **Supplementary Table 4**). 100 DAMs (47 upregulated and 53 downregulated metabolites) were identified between WT and *osseh1*under cold stress (WT-C vs *osseh1*-C; variable importance (VIP) scores ≥1 and T-test P<0.05; **Figure 2D**; **Supplementary Table 5**). These results suggested that *OsSEH1* regulate a broad range of the metabolite accumulation.

### Differentially regulated metabolites by *OsSEH1* under cold stress

**Figure 3:**
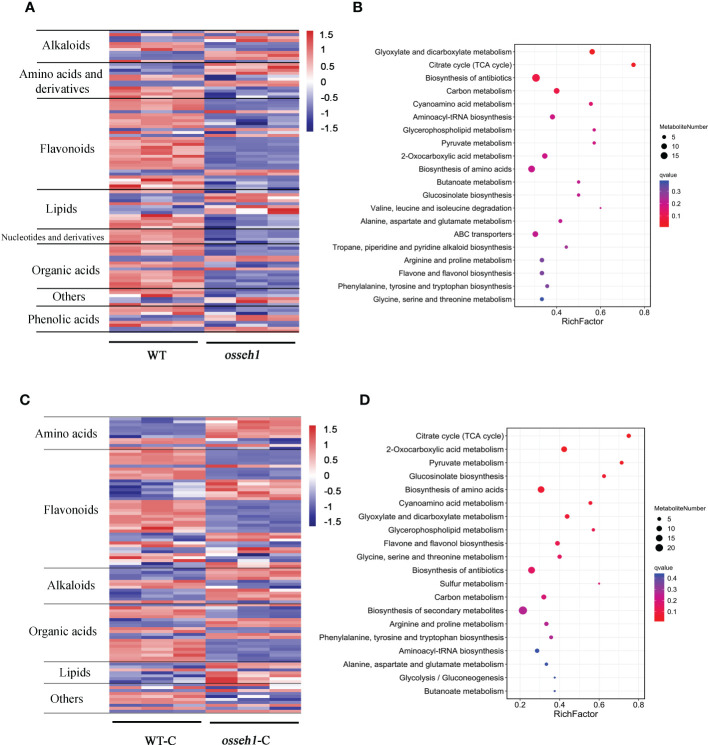
Differentially accumulated metabolites (DAMs) between *osseh1* lines and WT plants under normal condition and cold stress. **(A)** Heatmap of DAMs between *osseh1* lines and WT plants under normal condition. **(B)** The top 20 KEGG pathways of DAMs under normal condition. **(C)** Heatmap of DAMs between *osseh1* lines and WT plants under cold stress. **(D)** The top 20 KEGG pathways of DAMs under cold stress. WT, WT under normal condition. WT-C, WT under cold treatment. *osseh1*, *osseh1* lines under normal condition. *osseh1*-C, *osseh1* lines under cold treatment.

The DAMs between WT and *osseh1* lines under normal and cold condition were analyzed further. As expected, the metabolites expression patterns were similar between the biological replicates but differed significantly between the WT and *osseh1* mutant lines (**Figures 3A, C**). We detected the different accumulation pattern of a wide range of the amino acids, flavonoids, organic acids, alkaloids, phenolic acids and lipids between WT and *osseh1* mutants (**Figures 3A, C**). Kyoto Encyclopedia of Genes and Genomes (KEGG) pathway analysis demonstrated that glyoxylate and dicarboxylate metabolism, citrate cycle (TCA cycle) and biosynthesis of antibiotics were the most significantly changed pathways in the noncold treatment WT vs *osseh1* comparison (**Figure 3B**). However, the DAMs participating in citrate cycle (TCA cycle), 2-Oxocarboxylic acid metabolism, Pyruvate metabolism were mainly enriched under cold stress (**Figure 3D**). Further, some primary metabolic pathway that are essential for plant growth and development were also significantly enriched under cold stress, including biosynthesis of amino acids, flavone and flavonol biosynthesis and carbon metabolism. Flavonoids serve as ROS scavengers by locating and neutralizing radicals before they damage the cell thus important for plants (Agati et al., 2012). The contents of large-scale flavonoids were lower in *osseh1* mutants compared to WT plants under cold treatment (**Figure 3C**), suggesting that the *OsSEH1* may be involved in the regulation of flavone and flavonol biosynthesis.

### Overview of RNA-seq data analysis

**Figure 4:**
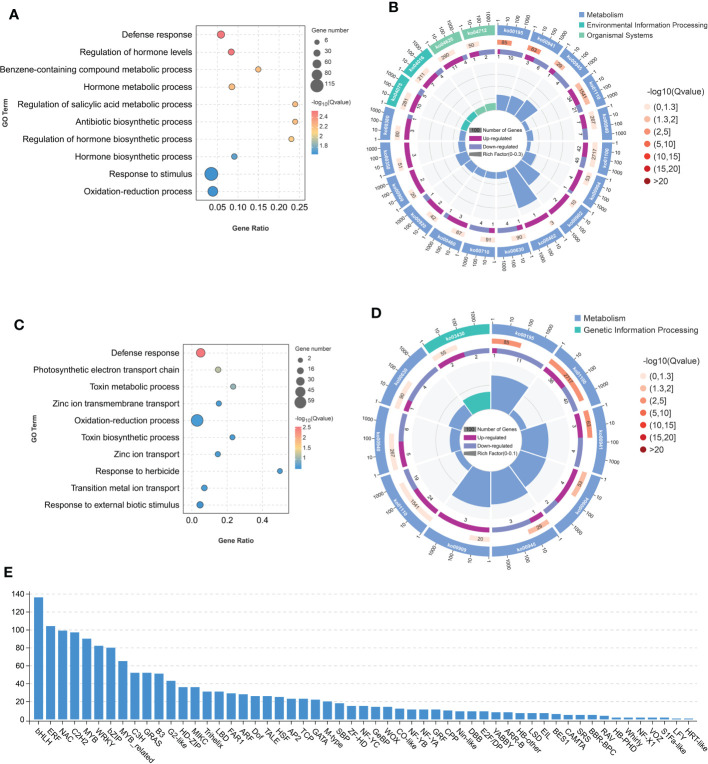
Differentially expressed genes (DEGs) between *osseh1* lines and WT plants under normal condition and cold stress. **(A)** The Gene Ontology (GO) enrichment of DEGs between *osseh1* lines and WT plants under normal condition. **(B)** The KEGG enrichment of the DEGs between *osseh1* lines and WT plants under normal condition. **(C)** The Gene Ontology (GO) enrichment of DEGs between *osseh1* lines and WT plants under cold stress. **(D)** The KEGG enrichment of the DEGs between *osseh1* lines and WT plants under cold stress. **(E)** Transcription factor analysis on the DEGs between *osseh1* lines and WT plants under cold stress.

We also performed a transcriptome assay using WT plants and *osseh1* mutants, in parallel with metabolomics. Under normal condition, we depicted 683 differently expressed genes (DEGs) that were up-regulated and 496 down-regulated (fold change > 2 or < 0.5, FDR<0.05; **Supplementary Figure 4A**; **Supplementary Table 6**) in the WT vs. *osseh1* comparison. Gene ontology (GO) analyses revealed that these DEGs were enriched in defense response, regulation of hormone levels, benzene-containing compound metabolic process, response to stimulus and oxidation-reduction process biological processes (**Figure 4A**). KEGG pathway analysis showed that photosynthesis (ko00195), flavonoid biosynthesis (ko00941) and stilbenoid, diarylheptanoid and gingerol biosynthesis (ko00945) were the most significantly changed pathways (**Figure 4B**).

Under cold condition, we depicted 514 differently expressed genes (DEGs) that were up-regulated and 429 down-regulated (fold change>2 or < 0.5, FDR<0.05; **Supplementary Figure 4B**; **Supplementary Table 7**) in the WT-C vs *osseh1*-C comparison. GO analyses revealed that these DEGs were enriched in multiple biological process, including defense response, photosynthetic electron transport chain, toxin metabolic process, zinc ion transmembrane transport and oxidation-reduction process (**Figure 4C**). KEGG pathway analysis demonstrated that photosynthesis (ko00195), metabolic pathways (ko01100) and flavonoid biosynthesis (ko00941) were the most significantly changed pathways in the WT-C vs *osseh1*-C comparison (**Figure 4D**). These results are consistent with our proposed role for *OsSEH1* in the regulation of cold stress tolerance.

To further understand the regulatory network of *OsSEH1* in the cold stress, we conducted transcription factor analysis on the differently expression genes between WT and *osseh1* lines. The results showed that significant changes in the expression level of many different types of transcription factors, including *bHLH* family, *ERF* family, *NAC* family, C2H2 family and MYB family (**Figure 4E**). The results indicated that OsSEH1 may regulate the expression of a large number transcription factors.

**Figure 5:**
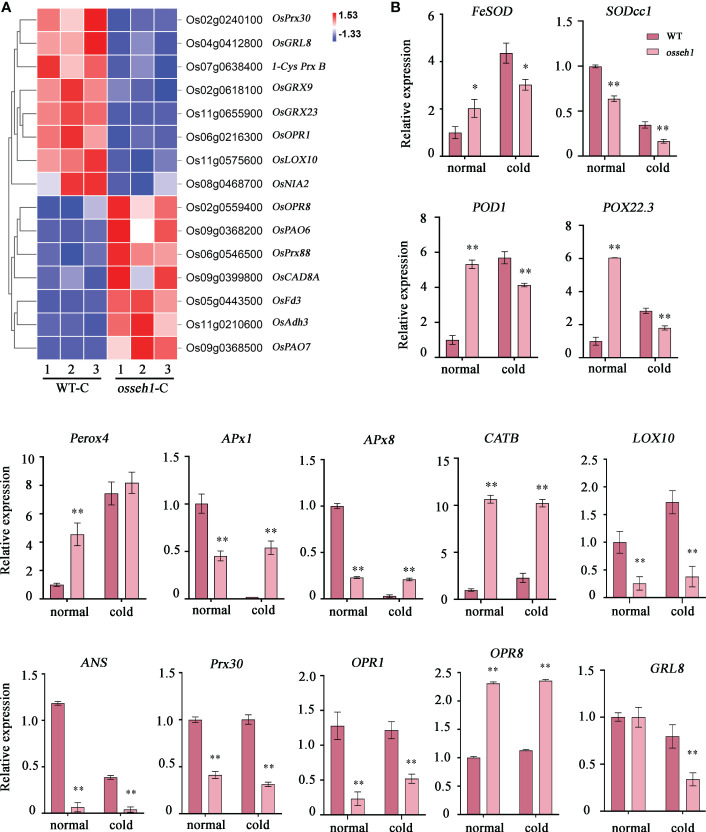
DEGs between *osseh1* knock-out lines and WT plants were involved in oxidation-reduction process. **(A)** Heatmap showing the enriched genes in the term of oxidation-reduction process. **(B)** Transcript levels of genes related to ROS scavenging in *osseh1* knock-out lines and WT plants under normal condition and cold stress. Data represents means ± SEM (n = 3). *P < 0.05, **P < 0.01. WT-C, WT under cold treatment. *osseh1*-C, *osseh1* lines under cold treatment.

In view of the differences in physiological characteristics between WT and *osseh1* lines, we also focused on the term of oxidation-reduction process. We found that a total of 59 DEGs involved in oxidation-reduction process, including multiple genes encoding oxidoreductase (**Figure 5A**; **Supplementary Table 8**). The result indicated a role of *OsSEH1* in the control of redox homeostasis. To confirm this possibility, we analyzed the expression levels of 14 genes by RT-qPCR (six genes in the DEGs analysis and eight other ROS-related genes) in WT and *osseh1* lines under normal condition and cold stress (**Figure 5B**). Of the 14 tested genes, *FeSOD*, *SODcc1*, *POD1*, *POX22.3, LOX10, ANS, Prx30, OPR1 and GRL8* were significantly lower in *osseh1* lines than in WT plants under cold stress, while the expression of *APx1*, *APx8*, *CATB* and *OPR8* were significantly higher in the *osseh1* lines than WT plants and the expression of *Perox4* was not significantly different between the *osseh1* lines and WT plants.

### Transcriptomic and metabolic profile of phenylpropanoid biosynthesis modulated by *OsSEH1*

**Figure 6:**
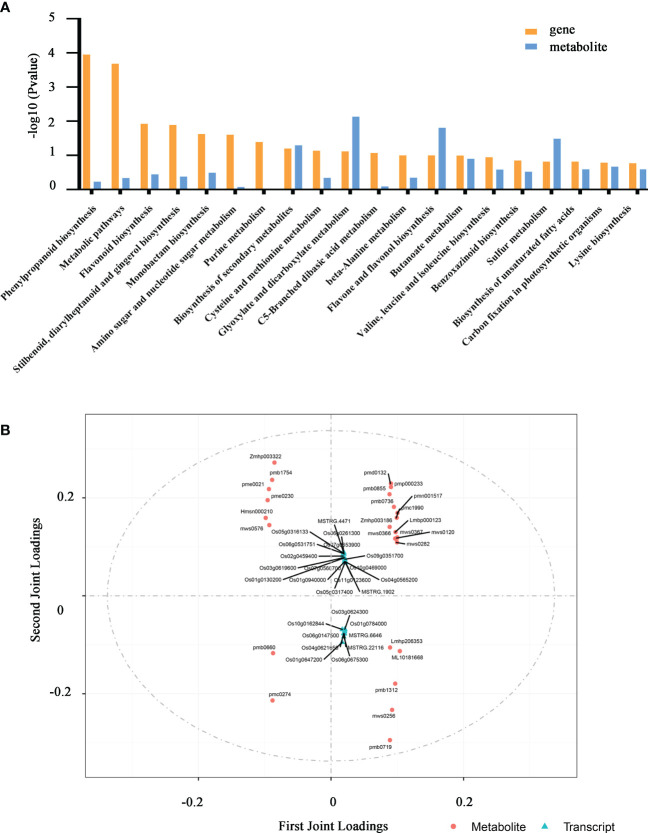
Combined analysis of the transcriptome and metabolome. **(A)** KEGG enrichment analysis of DAMs and DEGs between *osseh1* lines and WT plants under cold stress. **(B)** Loading values representation of genes and metabolites from transcriptome and metabolome Tables based on two-way orthogonal partial least squares (O2PLS) analysis.

To better characterize the role of *OsSEH1* in regulating genes and metabolites under cold stress, we conducted Pearson’s correlation analysis based on the transcriptomic and metabolomic data. The KEGG analysis of the correlated DEGs and DAMs showed that phenylpropanoid biosynthesis, metabolic pathways and flavonoid biosynthesis were the most enriched pathway under cold treatment (**Figure 6A**). Further, we performed an integrated Two-way Orthogonal Partial Least Squares (O2PLS) analysis of the transcriptome and metabolome Tables (**Figure 6B**). Consistent with the result of KEGG analysis, 11 of the top 25 metabolites were highly correlated with phenylpropanoid biosynthesis and flavonoid biosynthesis, including Isovitexin-2’’-O-(6’’’-p-coumaroyl) glucoside (Zmhp003322), Isovitexin-2’’-O-(6’’’-feruloyl) glucoside (Zmhp003186), Swertiajaponin (pmp000233), 4’-Hydroxy-5,7-dimethoxyflavanone (pmc1990), Tricin-4’-O-(guaiacylglycerol) ether-7-O-glucoside (pmb1312), Tricin-7-O-Glucoside (pmb0736), Tricin-4’-O-(syringyl alcohol) ether-5-O-glucoside (pmb0719), Isoorientin-7-O-(6’’-p-coumaroyl) glucoside (pmb0660), Tricin-4’-O-glucoside (Lmhp206353), L-Phenylalanine (pme0021) (**Figure 6B**).

**Figure 7:**
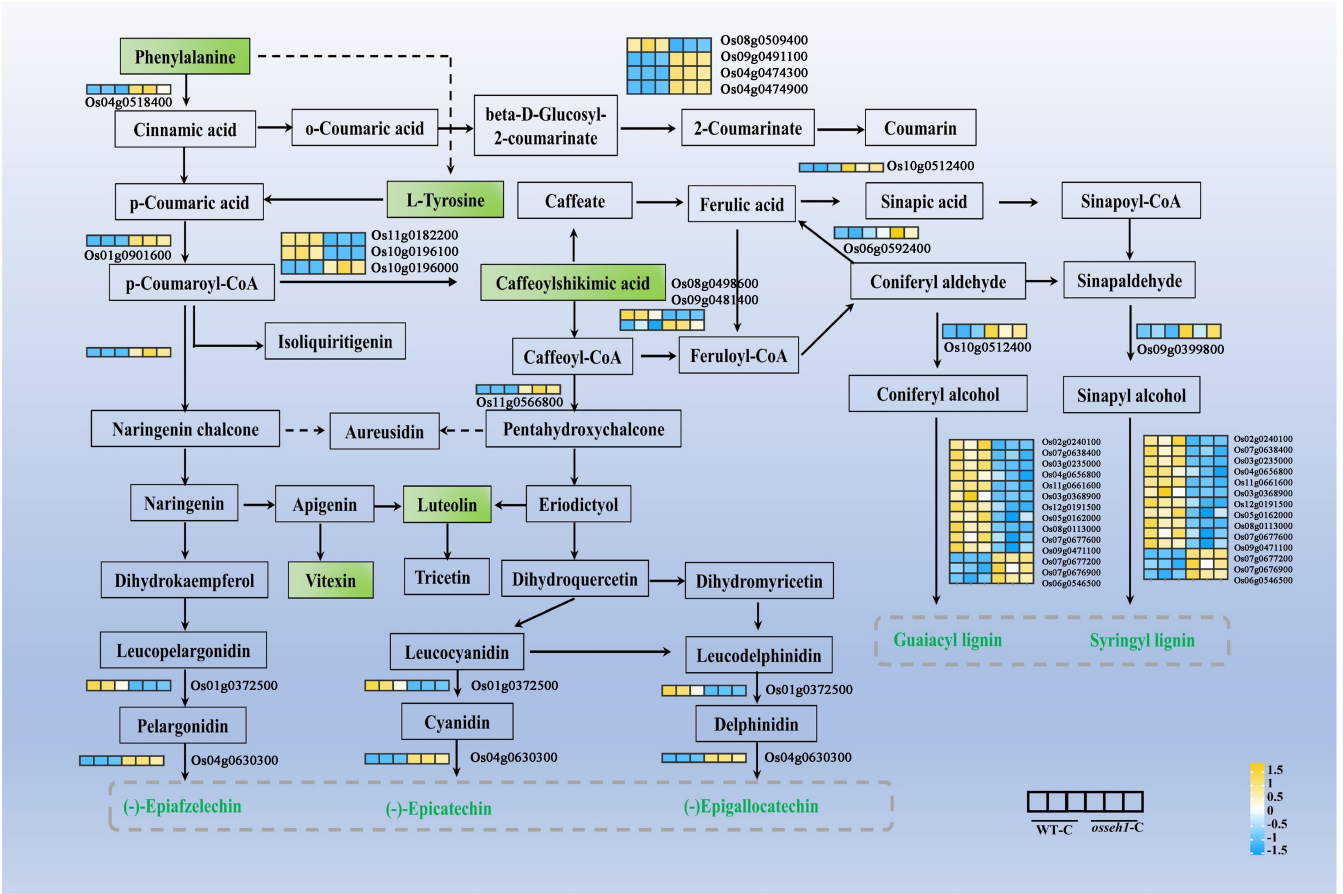
Different accumulation and expression patterns of metabolites and genes related to the phenylpropanoid biosynthesis pathway. Rectangle in the pathway indicates metabolite. The differentially accumulated metabolites are shown in green. The expression levels of genes are shown from yellow to blue (high to low) in the comparison of WT-C vs. *osseh1*-C. Gene heatmap shows the value of Log 2 (FPKM) in WT (left panel) and *osseh1* knock-out lines (right panel). WT-C, WT under cold treatment. *osseh1*-C, *osseh1* lines under cold treatment.

Moreover, we screened DEGs and DAMs in relation to their corresponding positions in the phenylpropanoid biosynthesis pathway. Metabolite analysis showed that (S)-alpha-Amino-beta-phenylpropionic acid (pme0021), Tyrosine (mws0250), 5-O-Caffeoylshikimic acid (Hmln002806), Apigenin 8-C-glucoside (mws0048) and 5,7,3’,4’-Tetrahydroxyflavone (pme0088) were all down-regulated in the *osseh1* lines compared with that of WT plants under cold treatment (**Figure 7**). Then, a total of 31 DEGs were identified in the phenylpropanoid biosynthesis pathway between the WT and *osseh1* lines under cold treatment. 16 genes encoding core players in the phenylpropanoid biosynthesis pathway, such as *PAL* (Os04g0518400), *ANS* (Os01g0372500), *4CL* (Os01g0901600), *prx30* (Os02g0240100), *prx38* (Os03g0235000), *prx45* (Os03g0368900), *prx58* (Os04g0656800), *prx72* (Os05g0162000), *prx115* (Os07g0677600) and *prx117* (Os08g0113000) were significantly down-regulated in *osseh1* lines compared with that in WT plants under cold stress (**Figure 7**). These evidences supported the conclusion that *OsSEH1* may play an essential role in the phenylpropanoid biosynthesis pathway in response to cold stress.

### Exogenous ABA increased the cold tolerance of *osseh1* knock-out lines

**Figure 8:**
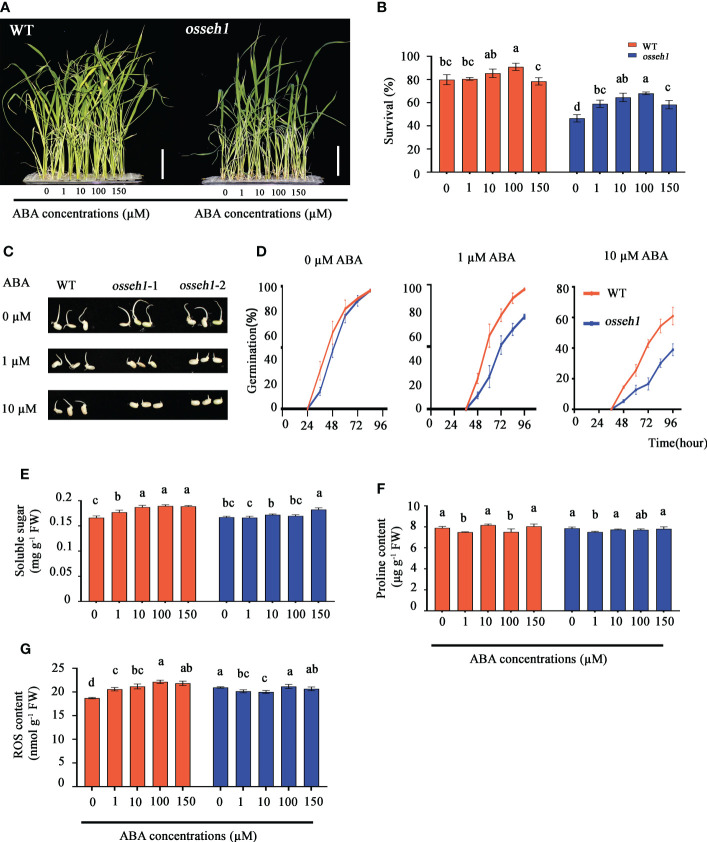
Responses of *osseh1* knock-out lines and WT plants to different concentration ABA under cold stress. **(A)** Phenotypes of *osseh1* knock-out lines and WT plants sprayed with different concentration ABA under cold stress. **(B)** Survival rates of *osseh1* knock-out lines and WT plants sprayed with different concentration ABA after cold treatment. **(C)** Germination phenotype of seeds from *osseh1* mutants and WT plants grown on 0, 1 or 10 µM ABA for 4 days after imbibition. **(D)** Germination rates corresponding to **(C)**. Statistical analysis of **(E)** soluble sugar, **(F)** proline content, **(G)** ROS content. Data represents means ± SEM (n = 3). Scale bars, 5cm.

The growth and adaptation to stress of plants are commonly regulated by multiple phytohormones. Meanwhile, we noticed that the DEGs between WT and *osseh1* knock-out lines were significantly enriched in regulation of hormone levels and hormone metabolic process (**Figure 4A**) and we thus speculated that *OsSEH1* may play a role in regulation of phytohormones. To verify our speculation, we separately sprayed exogenous indole-3-acetic acid (IAA), 2, 4-Epibrassinolide and abscisic acid (ABA) onto the leaves of WT and *osseh1* knock-out lines under cold stress. The cold tolerance of WT plants and *osseh1* knock-out lines sprayed with ABA was significantly improved compared with that without exogenous hormone, but not with IAA or 2, 4-Epibrassinolide. To test a potential connection between *OsSEH1* and ABA, we further determined that the survival of WT plants increased by 0.70%, 5.56%, 11.11%, -1.39% in the 1 μM, 10 μM, 100 μM and 150 μM ABA treatments, respectively, compared with that of the 0 μM ABA treatment, while those of *osseh1* lines were 12.5%, 18.06%, 21.54%, 11.81% respectively (**Figures 8A, B**). To further verify the sensitivity of WT and *osseh1* lines to exogenous ABA, we calculated the germination rates of seeds grown on 0 μM, 1 μM or 10 μM ABA. In the absence of ABA, there was no significant difference between *osseh1* mutants and WT plants (**Figures 8C, D**). However, the germination rates of *osseh1* seeds treated with 1 μM and 10 μM ABA were significantly inhibited compared with that of WT plants (**Figures 8C, D**). These results indicated that *osseh1* knock-out lines had ABA hypersensitive phenotypes compared with that WT lines.

Further, we measured soluble sugar content, proline content, ROS content, SOD activity, POD activity, APX activity and CAT activity in the leaves treated with different ABA concentrations of WT and *osseh1* lines. We measured the endogenous ABA content in the *osseh1* knock-out lines and WT plants to verify the effectiveness of exogenous ABA application. The endogenous ABA content of *osseh1* knock-out lines was significantly higher than that of WT plants before and after treatment (**Supplementary Figure 5A**). The content of soluble sugar in WT plants was increased as the ABA concentrations was increased, while it was almost no difference in *osseh1* lines under cold stress (**Figure 8E**). The trends of the proline content in both WT and *osseh1* lines showed no difference with the increase in ABA concentration (**Figure 8F**). The content of ROS in WT plants was increased as the ABA concentrations was increased, while it decreased significantly at the concentration of 1 μM and 10 μM in *osseh1* lines (**Figure 8G**). The activity of SOD in WT increased slowly as the ABA concentrations was increased, reaching a maximum with the spraying of 150 µM*L^-1^ ABA. In contrast, the activity of SOD in *osseh1* lines increase to a maximum with the spraying of 10 µM*L^-1^ ABA (**Supplementary Figure 5B**). Interestingly, in WT plants, the activity of POD was decreased as the ABA concentration was increased under cold stress, while it increased in *osseh1* plants (**Supplementary Figure 5C**). The activity of CAT and APX in both WT and *osseh1* plants increased with the increase in ABA concentration under cold stress (**Supplementary Figures 5D, E**). These results suggested that WT and *osseh1* knock-out plants differ in the sensitivity to exogenous ABA.

## Discussion

Low environmental temperature limits plant growth and development, so plants have evolved multiple response mechanism to adapt to cold stress. However, the mechanisms of perception and response to cold stress in rice remain largely unknown. Therefore, it is of interest to identify the cold-tolerant genes and their regulatory network in cold stress. In previous studies, we presented evidence that *OsSEH1* was a potential gene involved in the regulation of cold stress at the seedling stage (Song et al., 2018). In this study, we performed physiological, metabolomic and transcriptomic analyses of the leaves from *osseh1* knock-out lines and WT plants under normal and cold condition to understand the regulatory role in the cold stress of rice. Moreover, we provide several lines of evidence that *OsSEH1* functions in the oxidation-reduction process to regulate cold tolerance in rice. First, the content of ROS and the activity of antioxidant enzymes showed significant differences between *osseh1* knock-out lines and WT plants under cold stress (**Figures 1E–I**). Second, metabolomics analysis revealed that the contents of large-scale flavonoids serving as ROS scavengers were lower in *osseh1* mutants compared to wild type under cold treatment (**Figure 3C**). Finally, Transcriptome analysis revealed that the DEGs between *osseh1* knock-out lines and WT plants were enriched in oxidation-reduction process (**Figure 4C**).

Nuclear pore complex (NPC), located within invaginations of the nuclear envelope, is the key subcellular structure to ensure the normal working of nuclear function and cell activities. NPC connects cytoplasm and nucleoplasm serving as the only channel for the exchange of macromolecules (Meier and Brkljacic, 2009). NPC is composed of multiple copies of approximately 30 diverse proteins termed nucleoporins (Nups) (Rout et al., 2000; Zimmerli et al., 2021). Up to now, the research on the function of plant nucleoporin is mainly concentrated in Arabidopsis (Lee and Seo, 2015; Zhu et al., 2017; Zhang et al., 2020), and our knowledge on the functions of rice nucleoporins remains poor. In this study, the nucleoporin-encoding gene *OsSEH1* play a positive role in cold stress of rice as *osseh1* knock-out lines showed significant decreased survival rate compared with that of WT plants after cold treatment (**Figure 1A**). This study advances our understanding of the function of plant nucleoporins.

Multiple components of physiology and biochemistry, such as photosynthesis, respiration, enzymatic reactions, osmotic potential, secondary metabolism, and nutrient absorption are negatively impacted by cold stress on plants (Balabusta et al., 2016; Wang et al., 2021). As ROS are very sensitive to ambient temperature changes, cold stress usually causes rapid and excessive accumulation of ROS in the cells (Noctor and Foyer, 1998; Xia et al., 2015; Choudhury et al., 2017). To protect the plant cells from oxidative stress and maintain normal cell functions, plants scavenge the excess ROS through diverse antioxidant enzymes, such as SOD, POD, CAT and APX. Interestingly, we noticed that the soluble sugar content, ROS content, CAT activity and APX activity of WT were decreased after cold stress in this study. Further, we found similar results of soluble sugar content (Wang et al., 2022; Xu et al., 2023), ROS content (Wang et al., 2022; Shu et al., 2023) and antioxidative enzyme activity (Wang et al., 2022, Liu et al., Hao et al., 2022; Liu et al., 2022) have been reported in previous studies. This results probably due to the impairment of the physiological metabolic sites of soluble sugar, ROS, CAT and APX by cold stress (Hao et al., 2022). In addition, the soluble sugar content, ROS content, CAT activity and APX activity of plants fluctuated under cold stress (Wang et al., 2022; Xu et al., 2023; Shu et al., 2023), rather than continuous increasing or decreasing, resulting in decreased content or activity of the physiological characteristics in a period of time after cold stress.

Numerous studies have demonstrated that plant response to abiotic stresses by alleviating oxidative stress (Ning et al., 2010; Fang et al., 2015; Zhou et al., 2018; Xiong et al., 2018; Liu et al., 2018; Zhao et al., 2020). For example, overexpression of an *ERF* family transcription factor (TF), *OsLG3*, increases drought tolerance by participating in H_2_O_2_ homeostasis (Xiong et al., 2018). Increasing the expression of *OsLPTL159* enhances rice cold tolerance by minimizing the toxic effects of ROS (Zhao et al., 2020). In this study, the activity of SOD and APX showed a significant difference between *osseh1* knock-out lines and WT plants under cold stress (**Figures 1F, I**). In addition, RNA-seq data analysis showed multiple genes involved in oxidation-reduction process showed different expression levels between *osseh1* knock-out lines and wild type under cold stress (**Figure 5A**). Finally, the accumulation of amino acids, alkaloids, organic acids and lipids showed significant differences between the WT plants and *osseh1* mutants, suggesting an imbalance of antioxidation-related compounds metabolism in *osseh1* mutants (**Figures 3A, C**). These results demonstrated that *OsSEH1* improve cold tolerance may associated with reducing oxidative stress in cells by regulating the levels of redox genes and effectively scavenging ROS.

In plants, the content of phenylpropanoid and flavonoid metabolite are closely related to the ability to scavenge ROS under adverse environment (Agati et al., 2012). In this study, the results of integration of metabolomic and transcriptomic revealed that the phenylpropanoid and flavonoid biosynthetic pathway were significantly enriched (**Figure 6A**). Metabolome analysis suggested lower accumulation of phenylpropanoid metabolites was discovered in the leaves of *osseh1* knock-out lines compared with that of wild type plants after cold stress, such as phenylalanine, L-tyrosine, caffeoylshikimic acid, luteolin and vitexin (**Figure 7**). Phenylalanine, the starting component in the phenylpropyl biosynthesis pathway, is essential for all subsequent metabolic processes. Phenylalanine is transformed to t-cinnamic acid by PAL which is an important branch point enzyme regulated at the transcriptional level (Weitzel and Petersen, 2010). The biosynthesis of downstream metabolites, such as phenylpropanoid and flavonoid molecules, is impacted by the decreasing phenylalanine concentration. Some studies have demonstrated that the content of phenylpropanoid metabolites is mainly regulated at the transcriptional level (Liu et al., 2015; Dong and Lin, 2021). Our RNA-Seq data revealed that many genes involved in the phenylpropanoid biosynthesis pathway, including *PAL, ANS, 4CL, prx30, prx38, prx45, prx58, prx72, prx115, prx117* and *prx137*, showed different expression levels between *osseh1* knock-out lines and wild type plants at transcriptional level (**Figure 7**). These transcriptional and metabolic changes might indicate *OsSEH1* plays a role in the phenylpropanoid biosynthesis pathway to response to cold stress.

Plant hormones play important roles in the plant responses and resistance to multiple abiotic stresses. As a signal molecule against abiotic stress, ABA plays an important role in regulating multiple stress responses in plants (Li et al., 2020; Takahashi et al., 2020; Wang et al., 2020). Accumulated ABA increases the tolerance of drought stress by inducing closing of leaf stomata to reduce water loss from plants (Guajardo et al., 2016). ABA also increase the content of carbohydrates, ATP, NAD (H), and heat shock proteins to regulate heat stress response (Li et al., 2020). Previous studies indicated that an appropriate increased levels of ABA may be beneficial to improve cold tolerance of plants (Mega et al., 2015; Huang et al., 2016). Consistent with the previous studies, we determined in this study that low concentration of exogenous ABA did increase the cold tolerance of both *osseh1* knock-out lines and WT plants, while *osseh1* lines had more sensitive phenotypes than WT plants (**Figure 8**). However, the underlying mechanism of *OsSEH1* response to ABA still remains unclear.

In summary, we characterized a function of *OsSEH1* as a positive regulator of cold stress. Further, transcriptomic and metabolic profiling revealed that *OsSEH1* plays a role in the oxidation-reduction process by coordinately regulating genes expression and metabolite accumulation involved in phenylpropanoid and flavonoid biosynthetic pathway. In addition, *osseh1* lines had hypersensitive phenotypes to exogenous ABA compared with WT plants, suggesting that *OsSEH1* may mediate cold tolerance by regulating ABA levels. Considering the positive regulation of cold stress by *OsSEH1*, the manipulation of *OsSEH1* expression levels may be a powerful strategy to improve the tolerance to cold stress of plants.

## Data availability statement

The datasets presented in this study can be found in online repositories. The names of the repository/repositories and accession number(s) can be found below: https://ngdc.cncb.ac.cn/gsa/, CRA008530.

## Author contributions

MZ and DM conceived and designed the experiments. SG performed most of the experiments, analyzed the data and wrote the manuscript. JZ performed the physiology experiments. ZZ and WC performed the functional tests and RT-qPCR. HX analyzed the data. All authors contributed to the article and approved the submitted version.

## References

[B1] Agati G. Azzarello E. Pollastri S. Tattini M. (2012). Flavonoids as antioxidants in plants: Location and functional significance. Plant Sci. 196, 67–76. doi: 10.1016/j.plantsci.2012.07.014 23017900

[B2] Agati G. Brunetti C. Di Ferdinando M. Ferrini F. Pollastri S. Tattini M. (2013). Functional roles of flavonoids in photoprotection: New evidence, lessons from the past. Plant Physiol. And Biochem. 72, 35–45. doi: 10.1016/j.plaphy.2013.03.014 23583204

[B3] Balabusta M. Szafranska K. Posmyk M. M. (2016). Exogenous melatonin improves antioxidant defensein cucumber seeds (*Cucumis sativus* l.) germinated under chilling stress. Front. In Plant Sci. 7. doi: 10.3389/fpls.2016.00575 PMC4848318 27200048

[B4] Bates L. S. Waldren R. P. Teare I. D. (1973). Rapid determination of proline for water stress studies. Plant Soil 39, 305–307. doi: 10.1007/BF00018060

[B5] Boerjan W. Ralph J. Baucher M. (2003). Lignin biosynthesis. Annu. Rev. Plant Biol. 54, 519–546. doi: 10.1146/annurev.arplant.54.031902.134938 14503002

[B6] Choudhury F. K. Rivero R. M. Blumwald E. Mittler R. (2017). Reactive oxygen species, abiotic stress and stress combination. Plant J. 90, 856–867. doi: 10.1111/tpj.13299 27801967

[B7] Dong C.-H. Agarwal M. Zhang Y. Xie Q. Zhu J.-K. (2006a). The negative regulator of plant cold responses, HOS1, is a RING E3 ligase that mediates the ubiquitination and degradation of ICE1. Proc. Natl. Acad. Sci. United States America 103, 8281–8286. doi: 10.1073/pnas.0602874103 PMC1472463 16702557

[B8] Dong C.-H. Hu X. Tang W. Zheng X. Kim Y. S. Lee B.-H.. (2006b). A putative arabidopsis nucleoporin, AtNUP160, is critical for RNA export and required for plant tolerance to cold stress. Mol. Cell. Biol. 26, 9533–9543. doi: 10.1128/MCB.01063-06 17030626 PMC1698518

[B9] Dong N. Q. Lin H. X. (2021). Contribution of phenylpropanoid metabolism to plant development and plant-environment interactions. J. Of Integr. Plant Biol. 63, 180–209. doi: 10.1111/jipb.13054 33325112

[B10] Fang Y. J. Liao K. F. Du H. Xu Y. Song H. Z. Li X. H.. (2015). A stress-responsive NAC transcription factor SNAC3 confers heat and drought tolerance through modulation of reactive oxygen species in rice. J. Of Exp. Bot. 66, 6803–6817. doi: 10.1093/jxb/erv386 26261267 PMC4623689

[B11] Fecht-Christoffers M. M. Fuhrs H. Braun H.-P. Horst W. J. (2006). The role of hydrogen peroxide-producing and hydrogen peroxide-consuming peroxidases in the leaf apoplast of cowpea in manganese tolerance. Plant Physiol. 140, 1451–1463. doi: 10.1104/pp.105.070474 16489137 PMC1435823

[B12] Fujino K. Sekiguchi H. Matsuda Y. Sugimoto K. Ono K. Yano M. (2008). Molecular identification of a major quantitative trait locus, qLTG3-1, controlling low-temperature germinability in rice. Proc. Natl. Acad. Sci. United States America 105, 12623–12628. doi: 10.1073/pnas.0805303105 PMC2527961 18719107

[B13] Gaveliene V. Pakalniskyte L. Novickiene L. (2014). Regulation of proline and ethylene levels in rape seedlings for freezing tolerance. Cent. Eur. J. Of Biol. 9, 1099–1107. doi: 10.2478/s11535-014-0340-z

[B14] Guajardo E. Correa J. A. Contreras-Porcia L. (2016). Role of abscisic acid (ABA) in activating antioxidant tolerance responses to desiccation stress in intertidal seaweed species. Planta 243, 767–781. doi: 10.1007/s00425-015-2438-6 26687373

[B15] Guo X. Y. Liu D. F. Chong K. (2018). Cold signaling in plants: Insights into mechanisms and regulation. J. Of Integr. Plant Biol. 60, 745–756. doi: 10.1111/jipb.12706 30094919

[B16] Han Q.-H. Huang B. Ding C.-B. Zhang Z.-W. Chen Y.-E. Hu C.. (2017). Effects of melatonin on anti-oxidative systems and photosystem II in cold-stressed rice seedlings. Front. Plant Sci. 8. doi: 10.3389/fpls.2017.00785 PMC5425610 28553310

[B17] Han B. Ma X. D. Cui D. Wang Y. J. Geng L. Y. Cao G. L.. (2020). Comprehensive evaluation and analysis of the mechanism of cold tolerance based on the transcriptome of weedy rice seedlings. Rice 13, 14. doi: 10.1186/s12284-019-0363-1 32056019 PMC7018935

[B18] Hao L. L. Zhang Y. Li Y. Bai L. X. Yue D. F. Zhang H. Y.. (2022). Comprehensive comparative analysis and expression profiles and effects on physiological response of DEAD-box RNA helicase genes in lumnitzera littorea (Jack) voigt under cold stress. J. Of Plant Interact. 17, 595–607. doi: 10.1080/17429145.2022.2074158

[B19] Hassan S. Mathesius U. (2012). The role of flavonoids in root-rhizosphere signalling: opportunities and challenges for improving plant-microbe interactions. J. Of Exp. Bot. 63, 3429–3444. doi: 10.1093/jxb/err430 22213816

[B20] Huang L. Hong Y. B. Zhang H. J. Li D. Y. Song F. M. (2016). Rice NAC transcription factor ONAC095 plays opposite roles in drought and cold stress tolerance. BMC Plant Biol. 16. doi: 10.1186/s12870-016-0897-y PMC5029094 27646344

[B21] Ishitani M. Xiong L. Lee H. Stevenson B. Zhu J. K. (1998). HOS1, a genetic locus involved in cold-responsive gene expression in arabidopsis. Plant Cell 10, 1151–1161. doi: 10.1105/tpc.10.7.1151 9668134 PMC144054

[B22] Lee K. Seo P. J. (2015). The E3 ubiquitin ligase HOS1 is involved in ethylene regulation of leaf expansion in arabidopsis. Plant Signaling Behav. 10. doi: 10.1080/15592324.2014.1003755 PMC4622604 25848954

[B23] Lee H. Xiong L. Gong Z. Ishitani M. Stevenson B. Zhu J. K. (2001). The arabidopsis HOS1 gene negatively regulates cold signal transduction and encodes a RING finger protein that displays cold-regulated nucleo–cytoplasmic partitioning. Genes Dev. 15, 912–924. doi: 10.1101/gad.866801 11297514 PMC312662

[B24] Li P. He Q. Jin J. Liu Y. Wen Y. Zhao K.. (2022b). Tomato oxalyl-CoA synthetase degrades oxalate and affects fruit quality. Front. Plant Sci. 13. doi: 10.3389/fpls.2022.951386 PMC9301600 35874016

[B25] Liu G. C. Liu F. X. Wang Y. Liu X. (2022). A novel long noncoding RNA CIL1 enhances cold stress tolerance in arabidopsis. Plant Sci. 323. doi: 10.1016/j.plantsci.2022.111370 35788028

[B26] Liu J. Y. Osbourn A. Ma P. D. (2015). MYB transcription factors as regulators of phenylpropanoid metabolism in plants. Mol. Plant 8, 689–708. doi: 10.1016/j.molp.2015.03.012 25840349

[B27] Liu C. T. Ou S. J. Mao B. G. Tang J. Y. Wang W. Wang H. R.. (2018). Early selection of bZIP73 facilitated adaptation of japonica rice to cold climates. Nat. Commun. 9, 12. doi: 10.1038/s41467-018-05753-w 30120236 PMC6098049

[B28] Liu C. Schlaeppi M. R. Mao B. Wang W. Wang A. Chu C. (2019). The bZIP73 transcription factor controls rice cold tolerance at the reproductive stage. Plant Biotechnol. J. 17, 1834–1849. doi: 10.1111/pbi.13104 30811812 PMC6686130

[B29] Livak K. J. Schmittgen T. D. (2001). Analysis of relative gene expression data using real-time quantitative PCR and the 2(-delta delta C(T)) method. Methods (San Diego Calif.) 25, 402–408. doi: 10.1006/meth.2001.1262 11846609

[B30] Li C. Xu Y. Li Z. Cheng P. Yu G. (2022a). Transcriptomic and metabolomic analysis reveals the potential mechanisms underlying the improvement of β-carotene and torulene production in rhodosporidiobolus colostri under low temperature treatment. Food Res. Int. 156, 111158. doi: 10.1016/j.foodres.2022.111158 35651024

[B31] Li G. Y. Zhang C. X. Zhang G. H. Fu W. M. Feng B. H. Chen T. T.. (2020b). Abscisic acid negatively modulates heat tolerance in rolled leaf rice by increasing leaf temperature and regulating energy homeostasis. Rice 13, 16. doi: 10.1186/s12284-020-00379-3 32170463 PMC7070142

[B32] Lu G. Wu F.-Q. Wu W. Wang H.-J. Zheng X.-M. Zhang Y.. (2014). Rice LTG1 is involved in adaptive growth and fitness under low ambient temperature. Plant J. 78, 468–480. doi: 10.1111/tpj.12487 24635058

[B33] Ma Y. Dai X. Y. Xu Y. Y. Luo W. Zheng X. M. Zeng D. L.. (2015). COLD1 confers chilling tolerance in rice (vol 160, pg 1209, 2015). Cell 162, 222–222. doi: 10.1016/j.cell.2015.06.046 25728666

[B34] Mao D. H. Xin Y. Y. Tan Y. J. Hu X. J. Bai J. J. Liu Z. Y.. (2019). Natural variation in the HAN1 gene confers chilling tolerance in rice and allowed adaptation to a temperate climate. Proc. Of Natl. Acad. Of Sci. Of United States Of America 116, 3494–3501. doi: 10.1073/pnas.1819769116 PMC6397538 30808744

[B35] Ma Y. Y. Zhang Y. L. Lu J. Shao H. B. (2009). Roles of plant soluble sugars and their responses to plant cold stress. Afr. J. Of Biotechnol. 8, 2004–2010.

[B36] Mega R. Meguro-Maoka A. Endo A. Shimosaka E. Murayama S. Nambara E.. (2015). Sustained low abscisic acid levels increase seedling vigor under cold stress in rice (*Oryza sativa* l.). Sci. Rep. 5. doi: 10.1038/srep13819 PMC4563555 26350634

[B37] Meier I. Brkljacic J. (2009). The nuclear pore and plant development. Curr. Opin. In Plant Biol. 12, 87–95. doi: 10.1016/j.pbi.2008.09.001 18938103

[B38] Mittler R. (2017). ROS are good. Trends In Plant Sci. 22, 11–19. doi: 10.1016/j.tplants.2016.08.002 27666517

[B39] Moura J. Bonine C. A. V. Viana J. D. F. Dornelas M. C. Mazzafera P. (2010). Abiotic and biotic stresses and changes in the lignin content and composition in plants. J. Of Integr. Plant Biol. 52, 360–376. doi: 10.1111/j.1744-7909.2010.00892.x 20377698

[B40] Nakabayashi R. Yonekura-Sakakibara K. Urano K. Suzuki M. Yamada Y. Nishizawa T.. (2014). Enhancement of oxidative and drought tolerance in arabidopsis by overaccumulation of antioxidant flavonoids. Plant J. 77, 367–379. doi: 10.1111/tpj.12388 24274116 PMC4282528

[B41] Ning J. Li X. H. Hicks L. M. Xiong L. Z. (2010). A raf-like MAPKKK gene DSM1 mediates drought resistance through reactive oxygen species scavenging in rice. Plant Physiol. 152, 876–890. doi: 10.1104/pp.109.149856 20007444 PMC2815886

[B42] Noctor G. Foyer C. H. (1998). ASCORBATE AND GLUTATHIONE: Keeping active oxygen under control. Annu. Rev. Plant Physiol. Plant Mol. Biol. 49, 249–279. doi: 10.1146/annurev.arplant.49.1.249 15012235

[B43] Parry G. (2015). The plant nuclear envelope and regulation of gene expression. J. Of Exp. Bot. 66, 1673–1685. doi: 10.1093/jxb/erv023 25680795

[B44] Polle A. Krings B. Rennenberg H. (1989). Superoxide dismutase activity in needles of Norwegian spruce trees (*Picea abies* L.)1. Plant Physiol 90, 1310–1315. doi: 10.1104/pp.90.4.1310 PMC1061888 16666928

[B45] Rout M. P. Aitchison J. D. Suprapto A. Hjertaas K. Zhao Y. Chait B. T. (2000). The yeast nuclear pore complex: composition, architecture, and transport mechanism. J. Cell Biol. 148, 635–651. doi: 10.1083/jcb.148.4.635 10684247 PMC2169373

[B46] Sasaki T. Burr B. (2000). International rice genome sequencing project: the effort to completely sequence the rice genome. Curr. Opin. Plant Biol. 3, 138–141. doi: 10.1016/S1369-5266(99)00047-3 10712951

[B47] Shi Y. T. Ding Y. L. Yang S. H. (2018). Molecular reculation of CBF sicnalinc in colc acclimation. Trends In Plant Sci. 23, 623–637. doi: 10.1016/j.tplants.2018.04.002 29735429

[B48] Shu P. Li Y. Xiang L. Sheng J. Shen L. (2023). SlNPR1 modulates chilling stress resistance in tomato plant by alleviating oxidative damage and affecting the synthesis of ferulic acid. Scientia Hortic. 307, 111486. doi: 10.1016/j.scienta.2022.111486

[B49] Song J. Y. Li J. Q. Sun J. Hu T. Wu A. T. Liu S. T.. (2018). Genome-wide association mapping for cold tolerance in a core collection of rice (*Oryza sativa* l.) landraces by using high-density single nucleotide polymorphism markers from specific-locus amplified fragment sequencing. Front. In Plant Sci. 9. doi: 10.3389/fpls.2018.00875 PMC6036282 30013584

[B50] Sperotto R. A. De Araujo A. T. Adamski J. M. Cargnelutti D. Ricachenevsky F. K. De Oliveira B. H. N.. (2018). Deep RNAseq indicates protective mechanisms of cold-tolerant indica rice plants during early vegetative stage. Plant Cell Rep. 37, 347–375. doi: 10.1007/s00299-017-2234-9 29151156

[B51] Sudheeran P. K. Feygenberg O. Maurer D. Alkan N. (2018). Improved cold tolerance of mango fruit with enhanced anthocyanin and flavonoid contents. Molecules 23. doi: 10.3390/molecules23071832 PMC6100212 30041447

[B52] Sun M. Shen Y. Chen Y. Wang Y. Cai X. Yang J.. (2022). Osa-miR1320 targets the ERF transcription factor OsERF096 to regulate cold tolerance *via* JA-mediated signaling. Plant Physiol 189, 2500–2516. doi: 10.1093/plphys/kiac208 PMC9342977 35522026

[B53] Takahashi Y. Zhang J. Hsu P.-K. Ceciliato P. H. O. Zhang L. Dubeaux G.. (2020). MAP3Kinase-dependent SnRK2-kinase activation is required for abscisic acid signal transduction and rapid osmotic stress response. Nat. Commun. 11. doi: 10.1038/s41467-019-13875-y PMC6940395 31896774

[B54] Treutter D. (2005). Significance of flavonoids in plant resistance and enhancement of their biosynthesis. Plant Biol. (Stuttgart Germany) 7, 581–591. doi: 10.1055/s-2005-873009 16388461

[B55] Vanacker Carver Foyer (1998). Pathogen-induced changes in the antioxidant status of the apoplast in barley leaves. Plant Physiol. 117, 1103–1114. doi: 10.1104/pp.117.3.1103 9662553 PMC34926

[B56] Vanholme R. De Meester B. Ralph J. Boerjan W. (2019). Lignin biosynthesis and its integration into metabolism. Curr. Opin. In Biotechnol. 56, 230–239. doi: 10.1016/j.copbio.2019.02.018 30913460

[B57] Verma S. Dubey R. S. (2003). Lead toxicity induces lipid peroxidation and alters the activities of antioxidant enzymes in growing rice plants. Plant Sci. 164, 645–655. doi: 10.1016/S0168-9452(03)00022-0

[B58] Vogt T. (2010). Phenylpropanoid biosynthesis. Mol. Plant 3, 2–20. doi: 10.1093/mp/ssp106 20035037

[B59] Wang W. X. Du J. Chen L. M. Zeng Y. J. Tan X. M. Shi Q. H.. (2021). Transcriptomic, proteomic, and physiological comparative analyses of flooding mitigation of the damage induced by low-temperature stress in direct seeded early indica rice at the seedling stage. BMC Genomics 22. doi: 10.1186/s12864-021-07458-9 PMC7952222 33706696

[B60] Wang Q. Yu F. Xie Q. (2020). Balancing growth and adaptation to stress: crosstalk between brassinosteroid and abscisic acid signaling. Plant Cell Environment 43, 2325–2335. doi: 10.1111/pce.13846 32671865

[B61] Wang H. Zhong L. Fu X. Huang S. Fu H. Shi X.. (2022). Physiological and transcriptomic analyses reveal the mechanisms of compensatory growth ability for early rice after low temperature and weak light stress. Plants 11, 2523. doi: 10.3390/plants11192523 36235390 PMC9570567

[B62] Weitzel C. Petersen M. (2010). Enzymes of phenylpropanoid metabolism in the important medicinal plant Melissa officinalis l. Planta 232, 731–742. doi: 10.1007/s00425-010-1206-x 20563822

[B63] Xia X.-J. Zhou Y.-H. Shi K. Zhou J. Foyer C. H. Yu J.-Q. (2015). Interplay between reactive oxygen species and hormones in the control of plant development and stress tolerance. J. Of Exp. Bot. 66, 2839–2856. doi: 10.1093/jxb/erv089 25788732

[B64] Xiong H. Y. Yu J. P. Miao J. L. Li J. J. Zhang H. L. Wang X.. (2018). Natural variation in OsLG3 increases drought tolerance in rice by inducing ROS scavenging. Plant Physiol. 178, 451–467. doi: 10.1104/pp.17.01492 30068540 PMC6130013

[B65] Xu H. Li J. Wang L. Li X. Liu Y. Wang X.. (2023). Integrated transcriptomic and metabolomics analysis reveals abscisic acid signal transduction and sugar metabolism pathways as defense responses to cold stress in argyranthemum frutescens. Environ. Exp. Bot. 205, 105115. doi: 10.1016/j.envexpbot.2022.105115

[B66] Yang Y. Wang W. Chu Z. Q. Zhu J. K. Zhang H. M. (2017b). Roles of nuclear pores and nucleo-cytoplasmic trafficking in plant stress responses. Front. In Plant Sci. 8. doi: 10.3389/fpls.2017.00574 PMC5388774 28446921

[B67] Yang F. Y. Zhang Y. S. Wen-Ke L. I. Hou-Quan L. Luo J. M. (2017a). Chilling damage comprehensive assessment methods for rice. Chin. J. Appl. Ecol. 28, 3281–3288. doi: 10.13287/j.1001-9332.201710.021 29692147

[B68] Yan H. Zheng W. Wang Y. Wu Y. Yu J. Xia P. (2022). Integrative metabolome and transcriptome analysis reveals the regulatory network of flavonoid biosynthesis in response to MeJA in camelliavietnamensis Huang. Int. J. Mol. Sci. 23, 9370. doi: 10.3390/ijms23169370 36012624 PMC9409299

[B69] Yoshida S. Forno D. A. Cock J. H. Gomez K. A. (1971). Laboratory manual for physiological studies of rice. Int. Rice Res. Institute.

[B70] Zhang Z. Y. Li J. J. Pan Y. H. Li J. L. Zhou L. Shi H. L.. (2017). Natural variation in CTB4a enhances rice adaptation to cold habitats. Nat. Commun. 8, 13. doi: 10.1038/ncomms14788 28332574 PMC5376651

[B71] Zhang J. Y. Luo W. Zhao Y. Xu Y. Y. Song S. H. Chong K. (2016). Comparative metabolomic analysis reveals a reactive oxygen species-dominated dynamic model underlying chilling environment adaptation and tolerance in rice. New Phytol. 211, 1295–1310. doi: 10.1111/nph.14011 27198693

[B72] Zhang A. Q. Wang S. Kim J. Yan J. P. Yan X. F. Pang Q. Y.. (2020). Nuclear pore complex components have temperature-influenced roles in plant growth and immunity. Plant Cell And Environ. 43, 1452–1466. doi: 10.1111/pce.13741 32022936

[B73] Zhang Y. T. Yang L. W. Hu H. L. Yang J. J. Cui J. B. Wei G. Q.. (2022). Transcriptome and metabolome changes in Chinese cedar during cold acclimation reveal the roles of flavonoids in needle discoloration and cold resistance. Tree Physiol. 42, 1858–1875. doi: 10.1093/treephys/tpac046 35451493

[B74] Zhao J. Wang S. S. Qin J. J. Sun C. Q. Liu F. X. (2020). The lipid transfer protein OsLTPL159 is involved in cold tolerance at the early seedling stage in rice. Plant Biotechnol. J. 18, 756–769. doi: 10.1111/pbi.13243 31469486 PMC7004919

[B75] Zhou Y. B. Liu C. Tang D. Y. Yan L. Wang D. Yang Y. Z.. (2018). The receptor-like cytoplasmic kinase STRK1 phosphorylates and activates CatC, thereby regulating H2O2 homeostasis and improving salt tolerance in rice. Plant Cell 30, 1100–1118. doi: 10.1105/tpc.17.01000 29581216 PMC6002193

[B76] Zhu Y. F. Wang B. S. Tang K. Hsu C. C. Xie S. J. Du H.. (2017). An arabidopsis nucleoporin NUP85 modulates plant responses to ABA and salt stress. PloS Genet. 13. doi: 10.1371/journal.pgen.1007124 PMC5741264 29232718

[B77] Zimmerli C. E. Allegretti M. Rantos V. Goetz S. K. Obarska-Kosinska A. Zagoriy I.. (2021). Nuclear pores dilate and constrict in cellulo. Science 374, 1341–134+. doi: 10.1126/science.abd9776 34762489

