# Supplementary material for: Multi-omics approach reveals the contribution of OsSEH1 to rice cold tolerance
Source: Front Plant Sci. 2023 Jan 13;13:1110724. doi: 10.3389/fpls.2022.1110724 (PMC9880419; doi:10.3389/fpls.2022.1110724)
Supplement: Supplementary file 1 [file Table_1.docx]

**A**

**
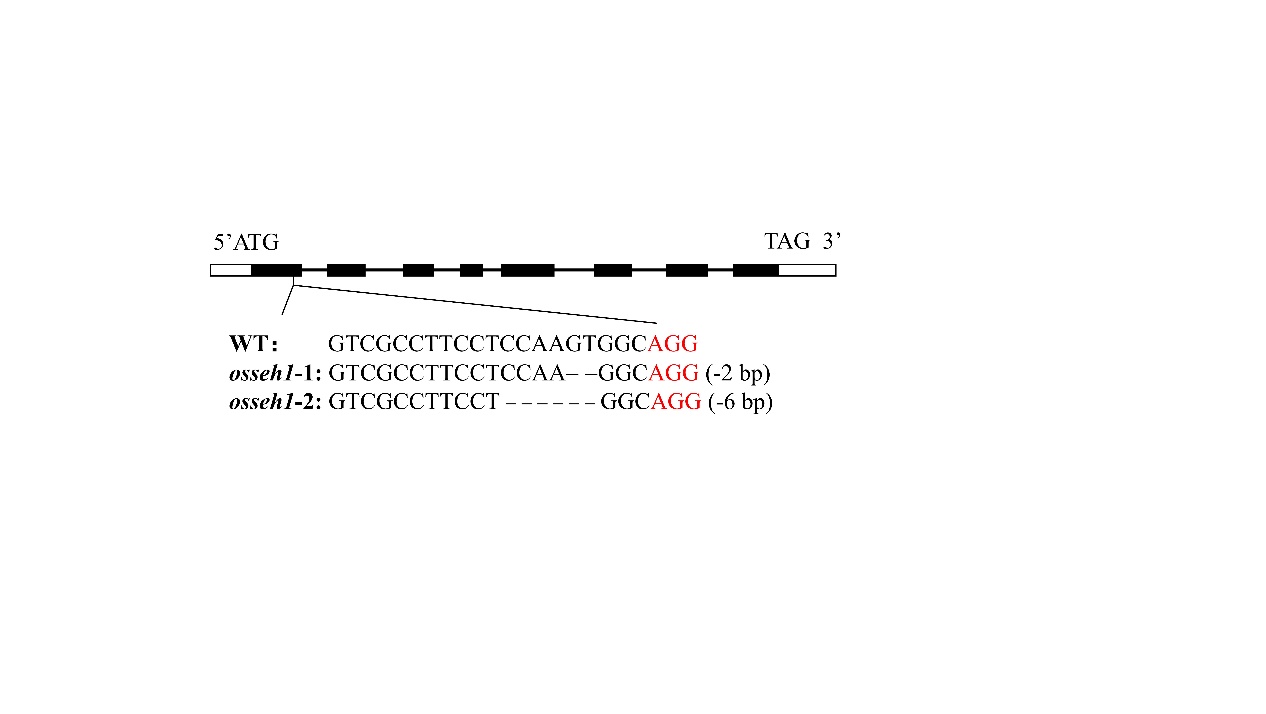
**

**B**

**
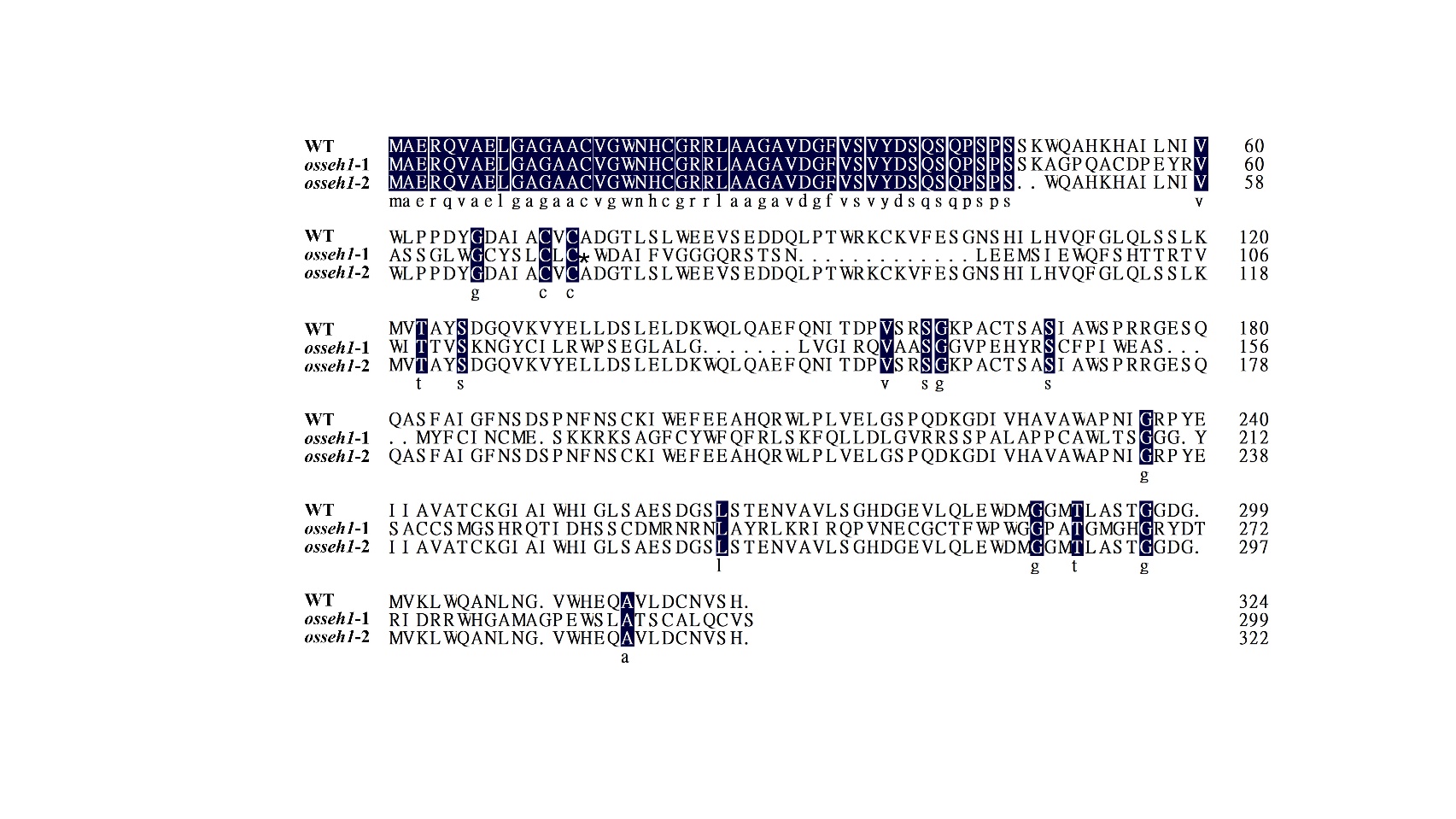
**

Supplementary Figure 1. (A) Sketch map of the mutation sites in the *osseh1* knockout line. The *osseh1* mutant with 2-bp or 6-bp deletion was obtained by CRISPR/Cas9. (B) The *osseh1*-1 mutant had two base pair deletion leading to a pre stop codon and the *osseh1*-2 mutant had two amino acids deletion.­­


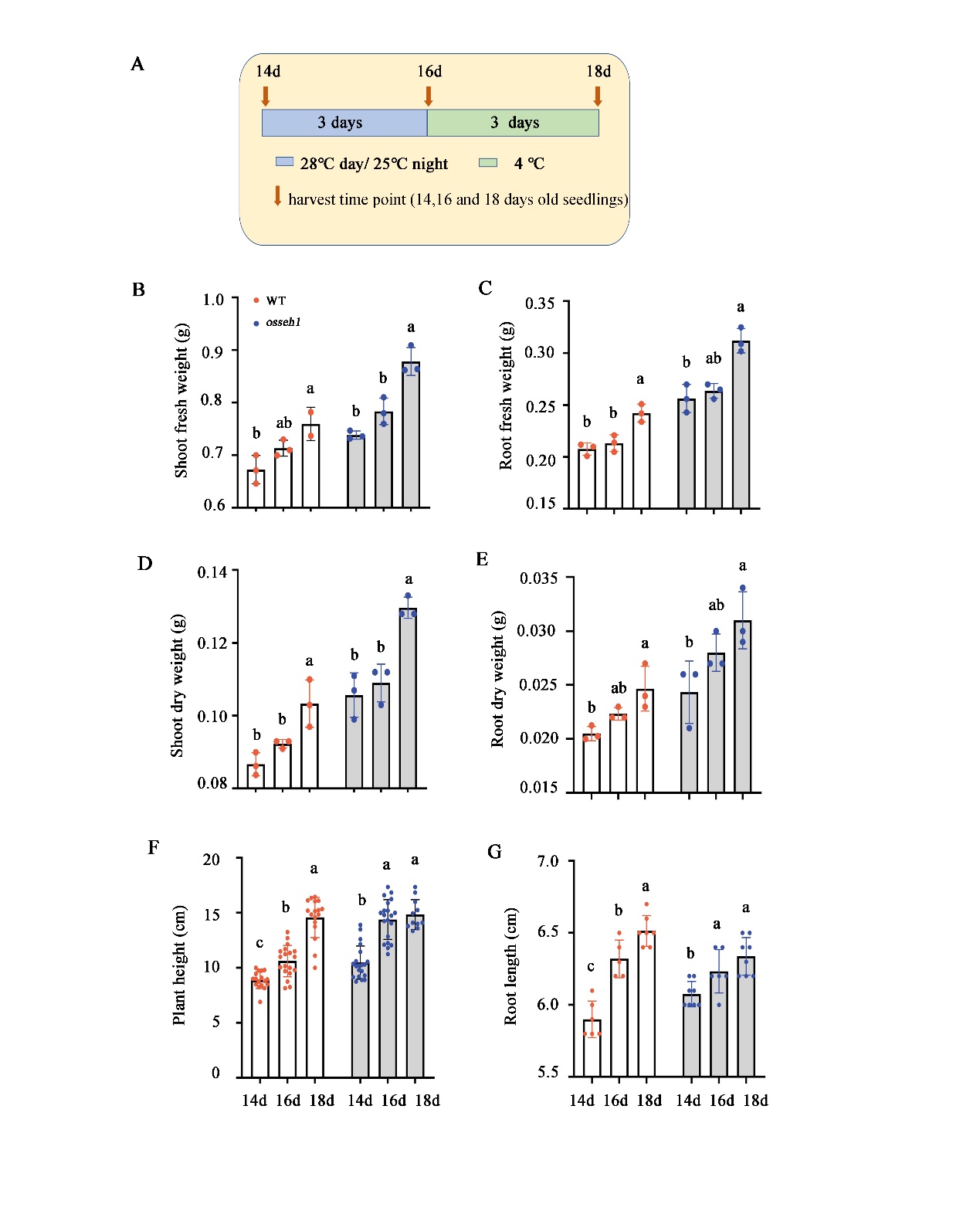


Supplementary Figure 2. Comparison of morphological indicators between *osseh1* knock-out lines and WT plants under cold stress. **(A)**Three sampling points for the determination of morphological indicators. Statistical analysis of **(B)** shoot fresh weight, **(C)** root fresh weight, **(D)** shoot dry weight, **(E)** root dry weight, **(F)** plant height, **(G)** root length. *P < 0.05, **P < 0.01.


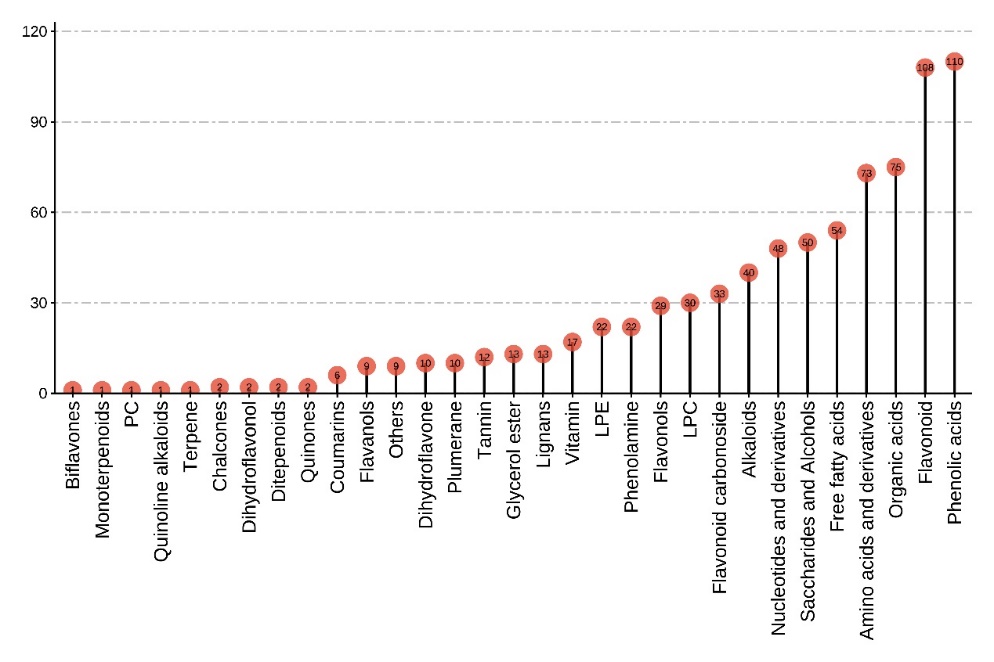


Supplementary Figure 3. Metabolites detected in the metabolomics assay.


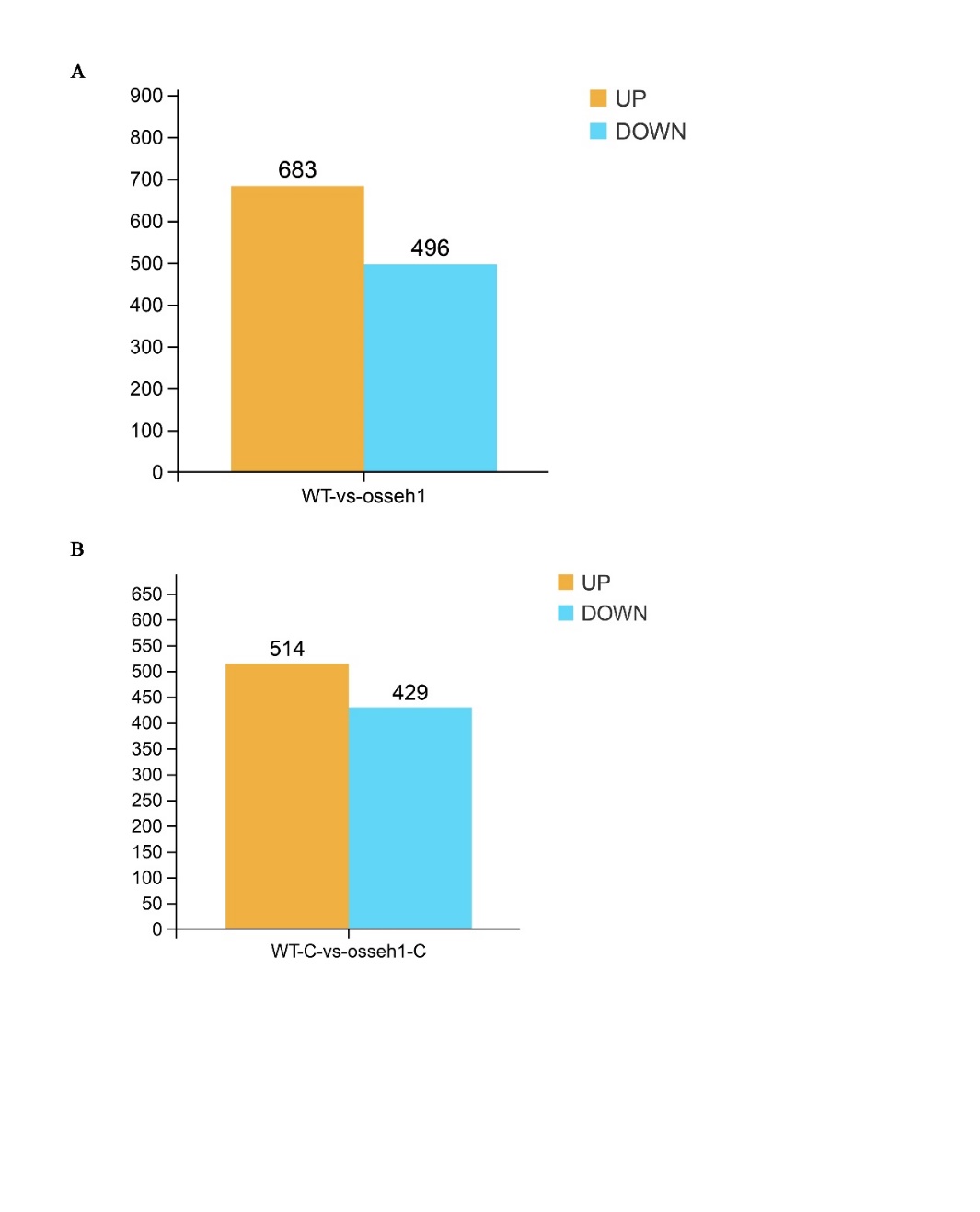


Supplementary Figure 4. The numbers of differentially expressed genes (DEGs) identified between *osseh1* knock-out lines and WT plants under normal and cold condition.

_
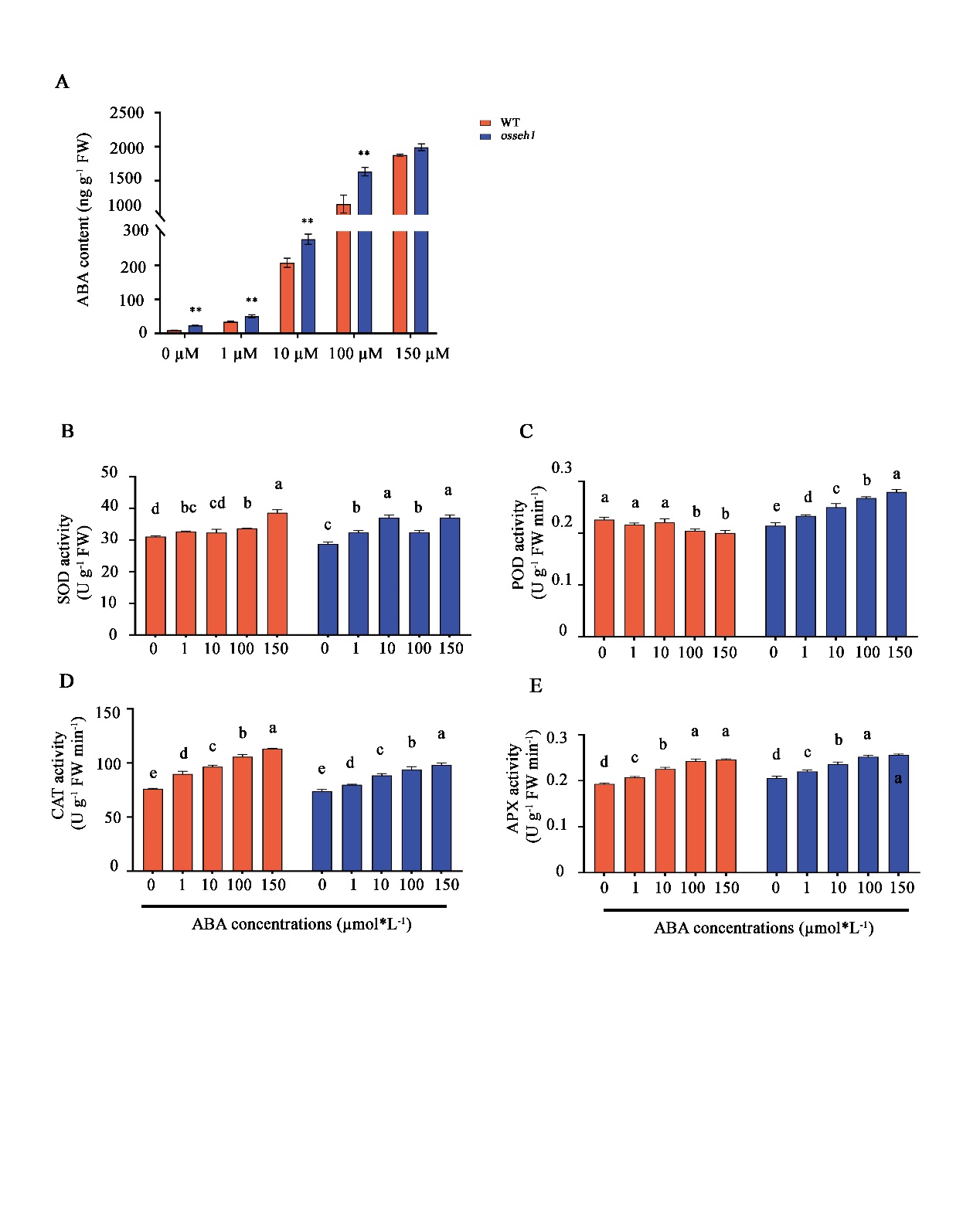
_

Supplementary Figure 5. Responses of *osseh1* knock-out lines and WT plants to different concentration ABA under cold stress. (A) ABA content in the leaves from *osseh1* knock-out lines and WT plants sprayed with different concentration exogenous ABA. Statistical analysis of (B) SOD activity, (C) POD activity, (D) CAT activity and (E) APX activity. Data represents means ± SEM (n = 3).
